# Root saprotropism: A novel tropism for navigating decay in the soil

**DOI:** 10.1016/j.xplc.2026.102077

**Published:** 2026-08-17

**Authors:** Yilin Du, Chunyan Wang, Gaojian Li, Zhaojun Ding

**Affiliations:** 1 The Key Laboratory of Plant Development and Environmental Adaptation Biology, Ministry of Education, School of Life Sciences, Shandong University, Qingdao, Shandong 266237, China

## Main text

As sessile organisms, plants have evolved sophisticated strategies to explore complex and heterogeneous soil environments. Root tropisms, directional growth responses to environmental stimuli, are essential for this subterranean navigation (Gilroy, 2008). Classical tropisms, including gravitropism, phototropism, hydrotropism, and halotropism, enable plants to anchor themselves in soil, forage for water and nutrients, and avoid adverse conditions (Whippo and Hangarter, 2006; Galvan-Ampudia et al., 2013; Dietrich, 2018; Roychoudhry and Kepinski, 2024). These responses are largely understood through the Cholodny–Went model, which posits that asymmetric auxin distribution drives differential cell elongation to generate bending (Roychoudhry et al., 2025). However, soil is not only a physical and chemical matrix. It is a biologically dynamic ecosystem shaped by shifting microbial communities. Microbial decomposition of dead plant material is ubiquitous in soils and drives nutrient recycling, simultaneously creating localized decay zones with intense microbial activity that may harbor pathogenic or spoilage-associated organisms. Whether living roots have evolved the capacity to actively sense and evade these potentially hostile underground niches has long been an outstanding question.

## Saprotropism: A conserved root avoidance response to decaying plant material

A new study by Bao and colleagues has shed light on this question by identifying “saprotropism,” a previously unrecognized root tropism that enables plants to bend away from decaying plant debris (Bao et al., 2026). The authors created localized decay zones using rotting apple debris in natural soil and controlled agar-plate systems. Direct contact with this decaying material strongly inhibited root growth and reduced plant fitness, whereas roots positioned nearby—but not in direct contact with the debris—consistently bent away from the source of decay. This directional avoidance response was observed in diverse species, including *Arabidopsis thaliana*, *Brassica napus*, *Solanum lycopersicum*, and *Triticum aestivum*, underscoring its broad biological significance. Intriguingly, saprotropism was specifically triggered by decaying plant material (apple, leaf, and sawdust) but not decaying animal material (chicken), suggesting that roots can discriminate between different types of decaying organic matter.

Subsequent multi-omics analyses demonstrated that fungi colonizing the decaying plant material—not bacteria—were the primary producers of acidic metabolites such as organic and phenolic acids. These acids diffused into the surrounding soil and established stable, long-lasting acidic pH gradients that served as directional cues. Using split-agar systems and pH-indicator staining, the authors showed convincingly that these decay-induced acidic gradients were both necessary and sufficient to guide root navigation. Neutralizing the decay-conditioned medium with potassium hydroxide or buffering it with MES abolished the bending response, whereas an artificial acidic pH gradient alone largely recapitulated saprotropic bending. Notably, the strength of this chemical cue scaled with the steepness of the gradient, providing further evidence that an acidic pH gradient was the principal directional cue.

## The epidermal RGF–RGFR module perceives external pH asymmetry

**Figure 1:**
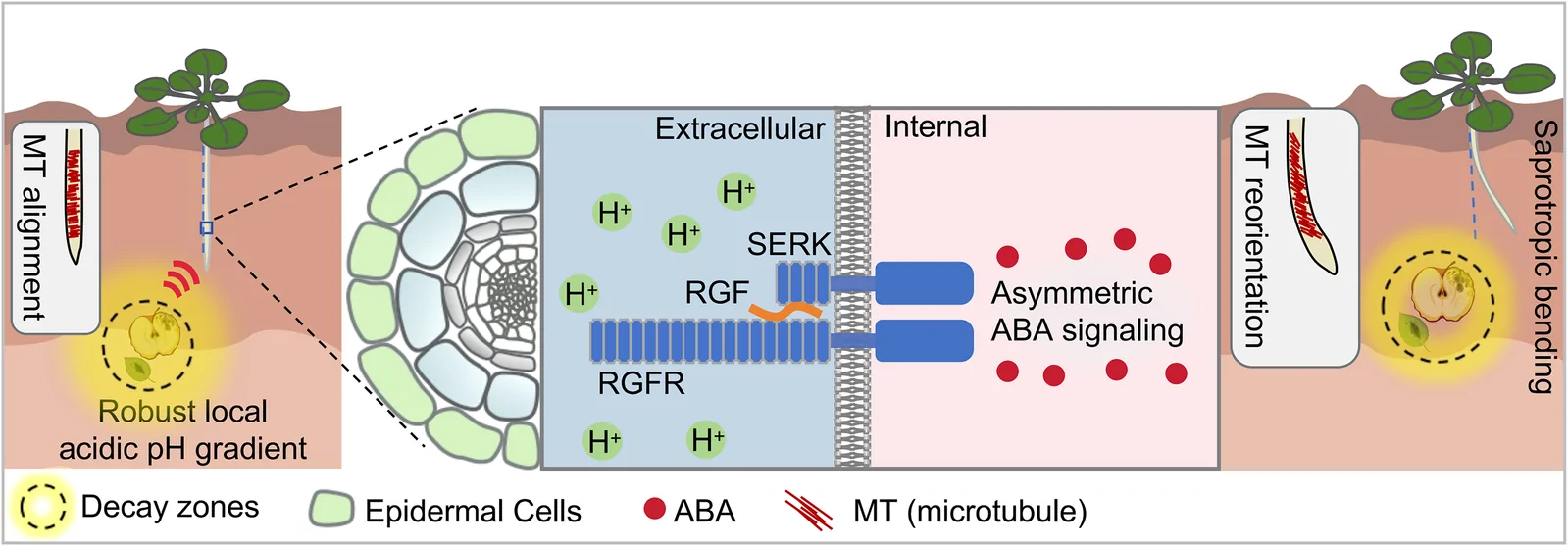
Roots exhibit saprotropic behavior, growing away from zones of microbial decay. The microbial decay process generates a local soil microenvironment characterized by stable acidic pH gradients. This pH asymmetry is sensed by an epidermal pH sensor, the RGF–RGFR peptide–receptor module, which translates the signal into an asymmetric distribution of ABA. This ABA asymmetry promotes microtubule-mediated anisotropic cell expansion and initiates handed root twisting, which together steer roots away from harmful decay zones. ABA, abscisic acid; MT, microtubule; RGF, root meristem growth factor; RGFR, root meristem growth factor receptor; SERK, somatic embryogenesis receptor-like kinase.

Strikingly, the authors found that neither asymmetric auxin nor cytokinin signaling underlies root saprotropism, indicating that this tropism operates independently of the classical Cholodny–Went model. Genetic screening revealed that the RGF–RGFR (ROOT MERISTEM GROWTH FACTOR–ROOT MERISTEM GROWTH FACTOR RECEPTOR) peptide–receptor module, which functions as an extracellular pH sensor (Liu et al., 2022), is essential for saprotropism. Loss-of-function mutants of *TPST* (tyrosylprotein sulfotransferase; required for RGF peptide sulfation) and the *RGFR* quadruple mutant (*rgi1/2/3/4*) displayed severe defects in root decay avoidance. Cell-type-specific rescue experiments demonstrated that expression of *TPST* exclusively in the root epidermal cells fully restored the response, whereas its expression in the cortex, endodermis, or root cap did not. Thus, the root epidermis serves as the primary site for perception of decay-induced pH asymmetry (Bao et al., 2026) (Figure 1).

## ABA asymmetry drives microtubule-dependent handed root twisting

Further investigation revealed that asymmetric distribution of abscisic acid (ABA) drives microtubule-dependent root twisting toward a decay source. Acting downstream of pH perception, the RGF–RGFR module transduces the external pH asymmetry into an internal hormonal cue. Using the advanced ABA biosensor nlsABACUS2-400n (Rowe et al., 2023), the authors visualized a rapid and pronounced asymmetric accumulation of ABA across the root, with elevated signals on the side facing the decay zone. Mutants defective in ABA biosynthesis (*nced3/5*), ABA perception (*pyl* duodecuple), or ABA signaling (*snrk2.2/2.3/2.6*) all exhibited dramatically impaired saprotropic bending. Crucially, epidermis-specific expression of the ABA signaling kinase SnRK2.2 rescued the defect in *snrk2.2/2.3* mutants, confirming that ABA signaling functions cell autonomously in the epidermis to transduce the external pH signal.

At the cellular level, this ABA asymmetry drives microtubule-dependent anisotropic cell expansion to promote the bending response. Confocal imaging of the microtubule marker *pTUB6*:mCherry-TUB6 revealed that decay stimulation induced a striking reorganization of cortical microtubules into oblique arrays within epidermal cells of the bending zone. This microtubule reorientation generated handed root twisting: left-side decay induced right-handed twisting and rightward bending, whereas right-side decay produced the mirror-image response (Yu et al., 2022). Both microtubule reorganization and handed cell twisting were abolished in *tpst-1, nced3/5, and snrk2.2/2.3/2.6 mutants*, demonstrating that the RGF–RGFR–ABA signaling axis controls saprotropic directionality via cytoskeletal remodeling (Bao et al., 2026) (Figure 1).

## Concluding remarks and perspectives

This study expands the current framework of plant tropisms in several respects. First, it establishes chemical gradients derived from microbial decay as bona fide environmental guidance cues, adding a new layer of complexity to root–soil–microbe communication. Second, saprotropism operates independently of the canonical Cholodny–Went auxin redistribution mechanism (Friml et al., 2002; Zhang et al., 2019); neither asymmetric auxin nor cytokinin signaling is required. In fact, auxin transport mutants displayed enhanced saprotropic bending, suggesting an antagonistic relationship between gravitropism and saprotropism, analogous to that between gravitropism and hydrotropism. This finding highlights how multiple tropisms are hierarchically integrated to coordinate root growth in complex environments.

In addition to these conceptual advances, the work of Bao and colleagues raises several key questions. One concerns the remarkable specificity of saprotropism for decaying plant matter rather than decaying animal matter: the molecular basis of this discrimination is unknown, in particular whether the RGF–RGFR complex shows intrinsic selectivity for plant-specific acidic metabolites or whether additional co-receptors/ligands are involved. Closely related is the issue of ligand–receptor complexity within the RGF peptide family. The relatively mild phenotype of the *rgf1/2/3* triple mutant versus the *rgi1/2/3/4* quadruple receptor mutant suggests substantial functional redundancy among RGF peptides. Higher-order genetic dissection will be required to define the complete ligand repertoire and to determine whether different RGF peptides respond to specific pH ranges or decay-derived chemical signatures.

A second major question involves the signal transduction cascade itself. Although the authors have demonstrated that the epidermal RGF–RGFR module perceives external pH asymmetry and that ABA then accumulates asymmetrically, the mechanism that connects these two events remains a critical missing link. Identification of the downstream signaling relays—whether involving G-protein activation, calcium signaling, or phosphorylation cascades—that translate pH asymmetry into hormonal asymmetry will be essential for characterizing the complete saprotropic signaling pathway and may reveal novel regulatory nodes unique to this tropic response.

From a broader perspective, saprotropism likely emerged after the transition to land, as aquatic environments would not support persistent, spatially resolved acidic gradients. Whether this tropism is conserved across all major land plant lineages, including bryophytes and early-diverging vascular plants, and how it contributed to terrestrial colonization remain open questions. Finally, artificial acidic pH gradients alone do not fully recapitulate the response induced by actual decay, suggesting that additional decay-derived metabolites (e.g., volatile organic compounds, phenolic derivatives, or microbial signals) may act together with pH gradients to fine-tune directional growth.

Addressing these questions will not only deepen our understanding of saprotropism as a novel tropic paradigm but also illuminate the broader principles governing how plants integrate physical, chemical, and biological cues from the soil environment to optimize root foraging and stress avoidance.

## Funding

This work was supported by the Taishan Scholar Special Project Fund of Shandong Province (tstp20231201) and the National Special Support Program for Leading Talents (2026WR01).

## Acknowledgments

No conflict of interest declared.

## Author contributions

Conceptualization, G.L. and Z.D.; visualization, G.L.; supervision, Z.D.; writing – original draft, Y.D., C.W., and G.L.; writing – review & editing, Z.D.
